# Interaction between Angiotensinase Activities in Pituitary and Adrenal Glands of Wistar–Kyoto and Spontaneously Hypertensive Rats under Hypotensive or Hypertensive Treatments

**DOI:** 10.3390/ijms22157823

**Published:** 2021-07-22

**Authors:** Ana B. Segarra, Isabel Prieto, Inmaculada Banegas, Magdalena Martínez-Cañamero, Ana B. Villarejo, Germán Domínguez-Vías, Marc de Gasparo, Manuel Ramírez-Sánchez

**Affiliations:** 1 Department of Health Sciences, University of Jaén, 23071 Jaén, Spain; asegarra@ujaen.es (A.B.S.); iprieto@ujaen.es (I.P.); ibanegas@ujaen.es (I.B.); canamero@ujaen.es (M.M.-C.); ab_villa@hotmail.com (A.B.V.); 2 Department of Physiology, Faculty of Health Sciences, Ceuta, University of Granada, 18071 Granada, Spain; germandv@ugr.es; 3 Cardiovascular & Metabolic Syndrome Adviser, Rue es Planches 5, 2842 Rossemaison, Switzerland; m.de_gasparo@bluewin.ch

**Keywords:** aminopeptidases, Wistar–Kyoto, spontaneously hypertensive rats, pituitary, adrenals, captopril, propranolol, L-NAME

## Abstract

In the present study, we analyzed the activity of several aminopeptidases (angiotensinases) involved in the metabolism of various angiotensin peptides, in pituitary and adrenal glands of untreated Wistar–Kyoto (WKY) and spontaneously hypertensive rats (SHR) or treated with the antihypertensive drugs captopril and propranolol or with the L-Arginine hypertensive analogue L-NG-Nitroarginine Methyl Ester (L-NAME). Intra- and inter-gland correlations between angiotensinase activities were also calculated. Membrane-bound alanyl-, cystinyl-, and glutamyl-aminopeptidase activities were determined fluorometrically using aminoacyl-β-naphthylamide as substrates. Depending on the type of angiotensinase analyzed, the results reflect a complex picture showing substantial differences between glands, strains, and treatments. Alanyl-aminopeptidase responsible for the metabolism of Ang III to Ang IV appears to be the most active angiotensinase in both pituitary and adrenals of WKY and particularly in SHR. Independently of treatment, most positive correlations are observed in the pituitary gland of WKY whereas such positive correlations are predominant in adrenals of SHR. Negative inter-gland correlations were observed in control SHR and L-NAME treated WKY. Positive inter-gland correlations were observed in captopril-treated SHR and propranolol-treated WKY. These results may reflect additional mechanisms for increasing or decreasing systolic blood pressure in WKY or SHR.

## 1. Introduction

**Figure 1:**
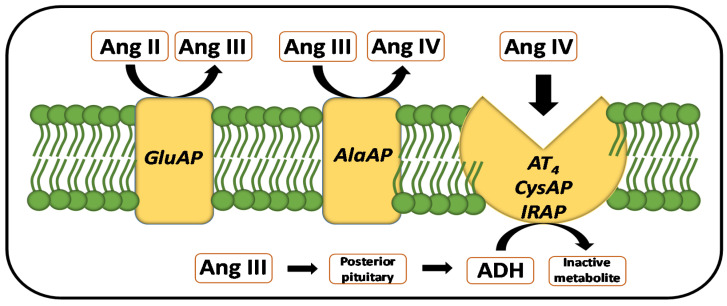
Partial representation of the renin-angiotensin system in which the hydrolytic and receptor function of the enzymatic activities (determined in the present work) is shown. Angiotensin II (Ang II) is hydrolyzed to Ang III by glutamate aminopeptidase (GluAP) and Ang III will be hydrolyzed to Ang IV by alanine aminopeptidase (AlaAP). Ang IV can bind to the AT_4_ receptor, reported as identical to the insulin-regulated aminopeptidase (IRAP) or vasopressinase, whose activity can be measured as cystine aminopeptidase (CysAP). In addition, the stimulatory role of Ang III on the posterior pituitary for antidiuretic hormone (ADH) secretion has been also reported [5,6,7,8,9].

The study of the renin-angiotensin system (RAS) remains one of the main objectives to better understand the pathogenesis of hypertension as well as to obtain suggestions for its treatment [1,2,3,4]. The analysis of angiotensinase activities, which metabolize angiotensin (Ang) peptides, allow the understanding of the dynamics of the functional status of their substrates as well as of the derived Ang peptides resulting from their activity [5] (Figure 1).

In short, focusing on the steps of the enzymatic cascade where the enzymes analyzed in this work act (Figure 1), Ang II is metabolized to Ang III by glutamate aminopeptidase (EC 3.4.11.7, GluAP) and Ang III is further metabolized to Ang IV by the action of alanine aminopeptidase (EC 3.4.11.2, AlaAP) [5]. Ang IV can bind to the AT_4_ receptor, which has been described to be identical to insulin-regulated aminopeptidase (IRAP) or vasopressinase, whose activity can be measured as cystine aminopeptidase (EC 3.4.11.3, CysAP) [6,7]. The binding of Ang IV to its AT_4_ receptor reduces vasopressinase activity and consequently the metabolism of vasopressin [8]. Furthermore, the stimulatory action of Ang III on the posterior pituitary for vasopressin secretion has also been described [9]. Wistar–Kyoto (WKY) and spontaneously hypertensive rats (SHR) are strains frequently used to study the role of RAS in hypertension as well as in its treatment [10,11].

Angiotensinase activities, involved in the metabolism of Ang peptides, are significantly and differently modified under several vasoactive drug treatments depending on the tissue considered such as brain regions, blood, kidney, or heart. Angiotensinases also interact significantly between these locations [12,13,14]. However, how they respond to vasoactive treatments and how the pituitary and adrenal neuroendocrine glands interact with these vasoactive treatments has not yet been reported. This knowledge may be important to better understand how the Ang metabolism is involved in the neuroendocrine response to treatments in normotensive and hypertensive subjects. Such knowledge may be important for the improvement of the treatment.

In the present study we analyzed the activity of several aminopeptidases (angiotensinases), involved in the metabolism of various angiotensin peptides, in pituitary (PT) and adrenal glands (AD) of untreated WKY and SHR or treated with the antihypertensive drugs captopril (CAP) and propranolol (PRO) or with the L-Arginine hypertensive analogue L-NAME (LN).

Our objective was to evaluate possible differences in enzymatic activities between the pituitary and adrenals in WKY and SHR as well as to study the probable influence of the different treatments on both glands and strains. Possible significant intra- and inter-gland interactions of enzymatic activities in WKY and SHR will also be analyzed.

In previous studies [7], it was proposed that the membrane-bound activity of angiotensinases acted more specifically at the tissue level than the soluble activity of these enzymes. Therefore, we focus on the analysis of the membrane-bound fraction in the present work. The findings for CysAP after captopril treatment and its interaction with water balance and systolic blood pressure (SBP) were previously reported in different tissues [7]. However, the interaction between different angiotensinase activities of PT and AD under diverse hypotensive and hypertensive treatments has not yet been analyzed.

It should be taken into account that the used methodology has several limitations. Indeed, each of the measured enzymatic activities may reflect the hydrolysis of various peptides. Therefore, the non-determination of the possible peptidergic substrates represents a limitation for the appropriate interpretation of the results. Furthermore, although the method used to obtain the membrane fraction is a validated standard method, the presence of specific membrane markers would have ensured the greater or lesser purity of the fraction.

## 2. Results

**Figure 2:**
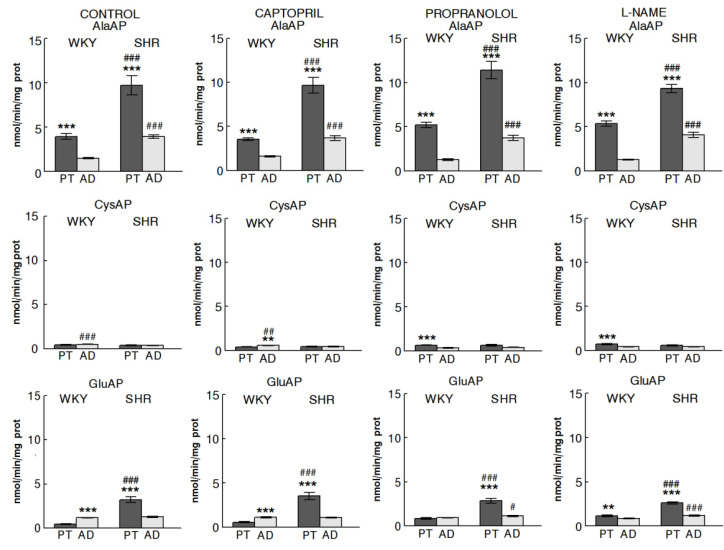
Regional differences of angiotensinase activities between pituitary (PT) and adrenal (AD) glands in Wistar–Kyoto (WKY) and spontaneously hypertensive rats (SHR). Bars represent Mean ± SEM levels of alanyl-aminopeptidase (AlaAP), cystinyl aminopeptidase (CysAP) and glutamyl-aminopeptidase (GluAP) activities, expressed as nmol/min/mg of proteins, in non-treated (control) or treated with captopril, propranolol or L-NAME. (*) differences between glands; (#) differences between strains for the same gland. Single sign (#, *p* < 0.05); double sign (**, ##, *p* < 0.01); triple sign (***, ###, *p* < 0.001).

**Figure 3:**
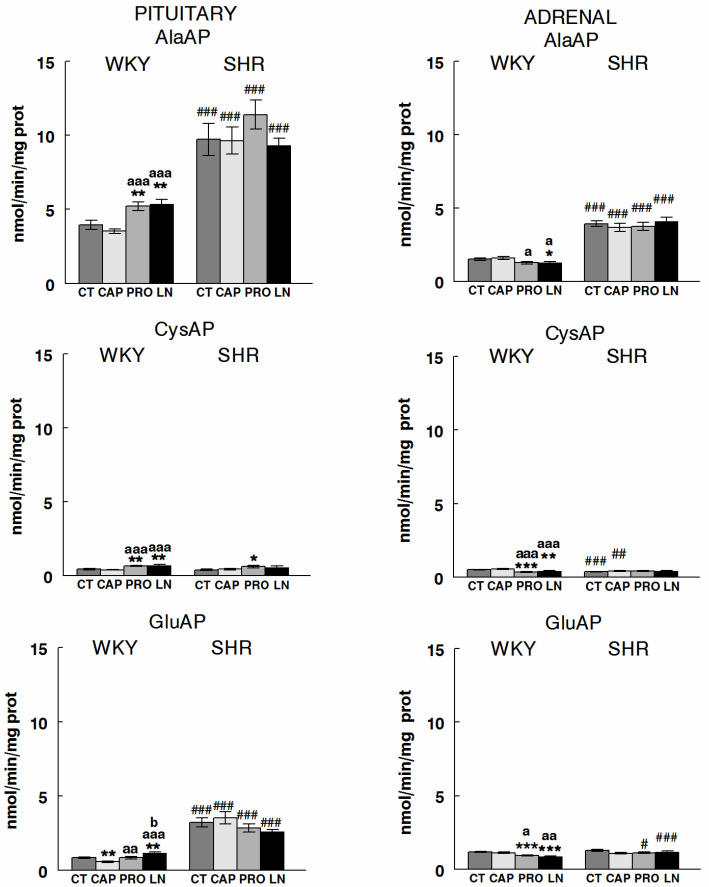
Influence of treatments on angiotensinase activities in pituitary (PT) and adrenal (AD) glands of Wistar–Kyoto (WKY) and spontaneously hypertensive rats (SHR). Bars represent Mean ± SEM levels of alanyl-aminopeptidase (AlaAP), cystinyl aminopeptidase (CysAP) and glutamyl-aminopeptidase (GluAP) activities, expressed as nmol/min/mg of proteins, in non-treated animals (control, CT) or treated with captopril (CAP), propranolol (PRO) or L-NAME (LN). (*) differences vs. control; (a) differences vs. CAP; (b) differences vs. PRO; (#) differences between strains. Single sign or letter (*, #, a, b, *p* < 0.05); double sign or letter (**, ##, aa, *p* < 0.01); triple sign or letter (***, ###, aaa, *p* < 0.001).

**Figure 4:**
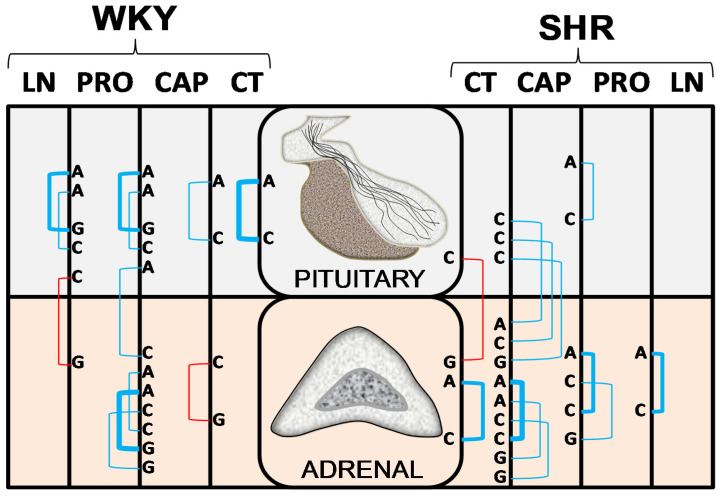
Significant intra- and inter-gland correlations between angiotensinase activities of pituitary and adrenal glands in non-treated Wistar–Kyoto (WKY) and spontaneously hypertensive rats (SHR) (control, CT) or treated with captopril (CAP), propranolol (PRO) or L-NAME (LN). Blue lines: positive significant correlations; red lines: negative correlations; line thickness: degree of significance (this representation corresponds to the values indicated in Table 1). Alanyl-aminopeptidase (A), cystinyl aminopeptidase (C) and glutamyl-aminopeptidase (G).

To facilitate the understanding of the results, Figure 2 is designed to highlight the regional response of the angiotensinases AlaAP, CysAP, and GluAP in control WKY and SHR and in animals treated with CAP, PRO, or LN. In addition, Figure 3 was designed to understand the influence of treatments more easily on WKY and SHR in both glands. Figure 4 reflects the significant intra- and inter-gland correlations in WKY and SHR in controls and under the different treatments. Systolic-, diastolic-blood pressure and heart rate were previously reported [12,15].

### 2.1. Regional Distribution

Systematically, AlaAP activity was higher in PT than AD and higher in SHR than in WKY. These results differ clearly from CysAP and GluAP activities. Comparing PT vs. AD in WKY, while CysAP was higher in AD under CAP treatment, it was higher in PT under PRO and LN treatments. However, no differences between glands were observed in SHR for CysAP activity. Considering GluAP activity, CT and CAP groups exhibited an opposite behavior when WKY and SHR were compared: while AD demonstrated higher activity than PT in WKY, it was higher in PT of SHR. Under PRO and LN treatments PT was higher than AD in SHR and in LN-treated WKY but no differences between glands were observed in WKY treated with PRO (Figure 2).

### 2.2. Influence of Treatments

Regarding the effect of treatments, AlaAP was not modified in SHR under any treatment neither in PT nor in AD. This behavior in SHR was the same for CysAP and GluAP where there were mainly no influence of treatments except for CysAP in PT in which PRO treatment exhibited significant (*p* < 0.05) higher levels than the CT group. However, in WKY, while PRO and LN increased AlaAP in PT, it decreased in AD with the same treatments. The results for CysAP were similar than AlaAP in WKY: higher activity under PRO and LN treatments in PT and lower in AD. Finally, the response of GluAP in WKY differs from the other activities as CAP treatment significantly reduced the activity in PT (*p* < 0.01) but not in AD (Figure 3).

### 2.3. Intra- and Inter-Gland Correlations

**Figure 5:**
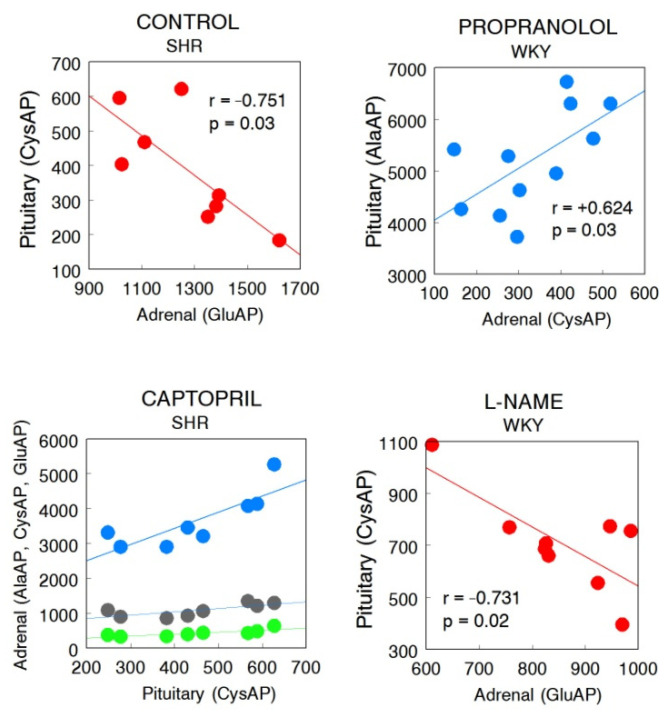
Inter-gland significant correlations obtained between the aminopeptidase activities analyzed in pituitary and in adrenals under the different treatments in WKY or SHR. In captopril-treated SHR: AD AlaAP vs. PT CysAP (blue) (r = +0.817, *p* = 0.01); AD CysAP vs. PT CysAP (charcoal) (r = +0.812, *p* = 0.01); AD GluAP vs. PT CysAP (green) (r = +0.737, *p* = 0.03).

Considering all WKY and SHR groups, significant correlations are mainly positives, especially intra-gland correlations in PT of WKY and AD of SHR. Considering treatments individually, a higher number of correlations were observed in WKY treated with PRO and in SHR treated with CAP (in both PT and AD) than the rest of treatments. Negative correlations raised always between CysAP and GluAP. Inter-gland correlations were observed in WKY LN and PRO and in SHR CT and CAP. No inter-gland correlations were observed in CT and CAP of WKY and in PRO and LN of SHR (Table 1 and Figure 4 and Figure 5).

Significant positive or negative (*italics*) correlations of membrane-bound aminopeptidase activities between pituitary and adrenals in the four groups studied in WKY and SHR. Pearson’s correlation coefficients (r) and *p* values are indicated. AlaAP, alanyl-aminopeptidase; CysAP, cystinyl-aminopeptidase; GluAP, glutamyl-aminopeptidase; PT, pituitary; AD, adrenals.

### 2.4. Correlations between SBP and Aminopeptidase Activities

**Figure 6:**
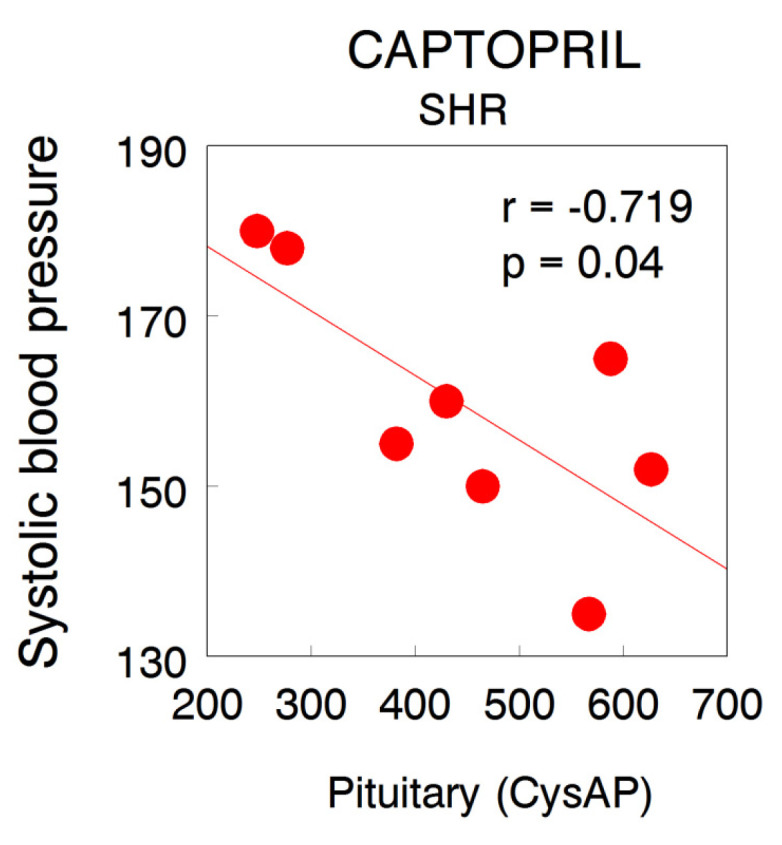
Level of correlation between systolic blood pressure (mmHg) and CysAP activity (nmol/min/mg of proteins) in the pituitary of captopril-treated spontaneously hypertensive rats.

Except for the correlation between pituitary CysAP and SBP levels in SHR under CAP treatment which achieved a slight but significant negative value (r = −0.719, *p* = 0.04) (Figure 6), no other significant correlations were observed between SBP and aminopeptidase activities at any location, treatment or strain.

## 3. Discussion

According to the results and following the angiotensin metabolism illustrated in Figure 1, the high levels of AlaAP in PT vs. AD and in SHR vs. WKY may suggest a higher formation of Ang IV in all groups studied. The results obtained for GluAP allow the suspicion of a higher formation of Ang III in the AD of WKY and in PT of SHR in controls and the same in CAP and PRO-treated animals, although no AD vs. PT differences were observed in PRO. However, while we could suggest a higher formation of Ang III in PT than AD in SHR after LN treatment, in contrast to CT, CAP, and PRO, the predominance of Ang III formation was observed in PT of WKY. Regarding CysAP, although a reduction in ADH could be suggested in the PT of PRO and LN, such ADH reduction was observed for AD in CAP (Figure 2). Considering the effect of treatments (Figure 3), while a higher formation of Ang IV could be suggested in the PT of WKY after PRO and LN treatment, a lower Ang IV would occur in the AD under the same treatments.

Systolic blood pressure levels for these WKY and SHR groups of animals were previously reported [12]. Briefly, in WKY rats while no significant differences were observed between CT, CAP, and PRO groups, a highly significant increase in SBP was observed for LN-treated animals, in comparison with the rest of groups. In SHR, SBP decreased significantly in animals treated with CAP in comparison with the rest of groups and while no differences were observed between CT and PRO groups, LN treatment increased SBP in comparison with all the groups. Following the action of the enzymes determined in the present work, we could suspect a higher or lower influence of the different peptides of the RAS cascade depending on the treatment (Figure 1). Therefore, regarding the significant correlations (Table 1, Figure 4 and Figure 5) and considering control groups, a highly significant intra-PT correlation was observed between AlaAP and CysAP in WKY rats which may suggest higher formation of Ang IV together with lower levels of ADH under basal normotensive conditions. In contrast, in CT SHR, there was a negative correlation between PT CysAP vs. AD GluAP. These results may suggest a higher inactivation of ADH in PT (or alternatively a higher function of the AT_4_ receptor), together with a lower formation of Ang III in the AD or vice versa, a lower inactivation of ADH in PT (or lower function of the AT_4_ receptor), and a higher formation of Ang III in the AD. In addition, CT SHR exhibited a highly significant positive correlation between AlaAP and CysAP in the AD: therefore, a higher formation of Ang IV may parallel a higher inactivation of ADH in the AD in basal hypertensive conditions.

In captopril groups, a non-significant reduction in SBP levels was observed in WKY under CAP treatment [12]. In this normotensive strain, a significant negative intra-AD correlation between CysAP and GluAP was observed. Interestingly, all the negative correlations involve CysAP vs. GluAP: the higher AD CysAP, the lower AD GluAP. This may indicate a lower ADH together with a higher Ang II in AD and vice versa. In CAP-treated SHR, all correlations were positive: the higher CysAP in PT, the higher AlaAP, CysAP, and GluAP in AD, suggesting a higher metabolism of ADH in PT and a higher metabolism of Ang II to Ang IV in AD. In addition, a highly significant positive correlation between AlaAP and CysAP was observed in AD. This may mean a higher Ang IV formation together with higher ADH inactivation in the AD. Interestingly, the character of all the correlations between aminopeptidase activities and SBP following CAP treatment in SHR was negative, and only a slight but significant correlation was achieved in the case of CysAP activity in PT (Figure 6). No other significant correlations were observed between SBP vs. aminopeptidase activities at any location, strain or treatment.

The mechanism by which CAP reduces SBP consists basically in its inhibitory effect on the angiotensin-converting enzyme thus reducing the formation of Ang II and interfering with its adrenergic potentiating effect in WKY and SHR. The blood pressure lowering effect of CAP is not totally clear: in fact, CAP reduces SBP in SHR but not in WKY [16] as we also observed previously [12]. Therefore, other mechanisms, such as differences in the response of the autonomic nervous system may be involved. The present results showing important differences in the levels of aminopeptidase activities and in the interaction between PT and AD in CAP groups of WKY and SHR, may be also considered in the mechanism of CAP action.

Propranolol treatment did not modify SBP neither in WKY nor in SHR [9]. All were intra-gland correlations in WKY as well as in SHR. The highest significant correlations in WKY were between AlaAP and GluAP in PT as well as in AD. These results suggest the activation of the metabolism from Ang II to Ang IV in PT and AD of WKY. In SHR there were positive correlations between AlaAP and CysAP in PT and AD. This suggests a lower Ang III and a higher Ang IV formation together with a higher metabolism of ADH in PT and AD.

In addition to the blockade of β-receptors, propranolol may exert its effects by other mechanisms such as the reduction of cardiac output, lower renin release, reduction of the sympathetic tone from the vasomotor center or a reduction of tyrosine-hydroxylase in adrenals [17]. These various mechanisms may affect the present results. We observe important differences between WKY and SHR in the response of aminopeptidases to PRO treatment as well as in the pattern of the correlations between both strains. This suggests again a different response depending on the behavior of the autonomic nervous system in WKY or SHR. Indeed, we have previously observed a divergent profile of response between hypothalamic and plasmatic aminopeptidase activities in WKY and SHR under PRO treatment, suggesting differences in the modulation of aminopeptidase activities by the sympathetic system [11]. The present results may also reflect additional mechanisms involved in PRO action.

Finally, after LN treatment, there was a highly significant intra-PT positive correlation between AlaAP and GluAP in WKY which may suggest a higher formation of Ang IV from the metabolism of Ang II. In this strain also appears a significant negative inter-gland correlation between PT and AD: the higher CysAP in PT (higher metabolism of ADH/higher AT_4_ receptor functionality) the lower GluAP in AD (lower metabolism/prolonged action of Ang II). In LN-treated SHR the intra-AD significant positive correlation between AlaAP and CysAP suggests higher Ang IV formation/lower AT_4_ function/higher ADH inactivation in AD.

The inhibition of nitric oxide synthase by L-NAME, leading to a reduction of nitric oxide and consequently to a decrease in its vasorelaxant function, is already an established model of experimental hypertension. However, this mechanism by itself does not explain the marked increase it produces in blood pressure. Therefore, other mechanisms have been suggested such as increased activity of the RAS as well as of the sympathetic nervous system [18]. Our results demonstrate marked changes in aminopeptidase activities, essentially in WKY rats but not in SHR and differences between WKY and SHR in the correlations between aminopeptidase activities under LN-treatment. This suggests an imbalance in the autonomic nervous system that could explain the differences between both strains.

Interestingly, if we only analyze the inter-gland significant correlations, they appeared in CT SHR, CAP-treated SHR, PRO-treated WKY and LN-treated WKY and it is always CysAP which was involved (Table 1, Figure 4 and Figure 5). In CT SHR, there was a negative correlation between PT CysAP vs. AD GluAP (the higher PT AT_4_/lower ADH, the lower GluAP/higher Ang II in the AD) which may reflect part of the deleterious influence of hypertension. In CAP-treated SHR, in which SBP decreased significantly in comparison with control hypertensive SHR rats [9], the positive correlations observed between PT CysAP activity and AD AlaAP, CysAP and GluAP activities suggest a higher AT_4_ function in PT together with higher Ang IV formation and vasopressin metabolism in the AD. These results might reflect an additional beneficial mechanism for CAP to reduce SBP. In PRO-treated WKY, in which SBP did not change in comparison with CT WKY [12], there was a slightly significant positive correlation between PT AlaAP and AD CysAP: the higher PT AlaAP (decreased Ang III, decreased ADH secretion/Increased Ang IV), the higher AD CysAP (increased vasopressin metabolism). In contrast to the previous three inter-gland correlations in CT SHR, PRO WKY, and CAP SHR, there was a negative significant interaction between PT CysAP and AD GluAP in LN-treated WKY, in which SBP increased significantly in comparison with normotensive CT WKY. This is suggesting a higher AT_4_ function in PT and lower Ang II metabolism in AD or vice versa: the lower CysAP in PT (lower degradation of ADH in PT), the higher Ang III formation in AD which may reflect the deleterious hypertensive effect of LN. These results may indicate additional mechanisms involved in the increase or decrease of SBP in WKY or SHR under hypotensive or hypertensive treatments.

It should be taken into account that in addition to angiotensin peptides and ADH, the peptidase activities determined in the present work also hydrolyze other peptidergic substrates such as enkephalins (AlaAP), oxytocin (CysAP) or cholecystokinin (GluAP) [1]. Therefore, in addition to the consequences of enzymatic activities on angiotensin metabolism, other supplementary effects could be expected such as their influence on opiates released from the pituitary or the adrenal gland and oxytocin from the pituitary.

## 4. Materials and Methods

### 4.1. Animals, Ethical Approval, and Drug Treatments

This study used 32 adult male WKY and 32 adult male SHR (Charles River Laboratories, Barcelona, Spain). Their weighs were between 100 and 150 g at the beginning of the experiments. WKY and SHR were randomly divided in four subgroups (*n* = 8 each): control (CT), captopril-treated (CAP), propranolol-treated (PRO) and L-NAME treated (LN). CAP (Sigma-Aldrich, St Louis, MO, USA; 100 mg/kg/day), PRO (Sigma-Aldrich, St Louis, MO, USA; 100 mg/kg/day) and LN (Sigma-Aldrich, St Louis, MO, USA; 70 mg/kg/day) were added to the drinking water of the respective subgroups for four weeks. The appropriate amount in the dose and the time of administration were previously indicated [19,20]. To avoid the influence of circadian variations and seasonal changes, the experiments were carried out between the months of April and July under constant light conditions between 9:00 a.m. and 12:00 p.m. [21]. All experiments were carried out in accordance with the European Communities Council Directive 86/609/EEC and was approved (code: SAF-2008-04685-C02-01; date: 9 January 2008) by the bioethics committee of the University of Jaén.

### 4.2. Blood Pressure Measurement

Systolic blood pressure was determined by tail-cuff plethysmography in unanesthetized rats (LE 5001-Pressure Meter; Letica SA, Barcelona, Spain) maintained in plastic holders at 37 °C. For the rats to adapt to the procedure, their pressure was measured several times during the treatment [22]. A minimum of 15 pressure measurements were carried out per rat, with the mean values within a range of 5 mmHg representing the recorded SBP level, not including the first and last determinations of each session.

### 4.3. Surgery and Tissue Samples

At the end of the treatment periods (four weeks) and once the SBP had been determined, the tissue samples were obtained under anesthesia with equithensin (2 mL/kg body weight) [42.5 g/L chloral hydrate dissolved in 19.76 mL ethanol, 9.72 g/L Nembutal (Sigma-Aldrich, St Louis, MO, USA) 0.396 L/L propylenglycol and 21.3 g/L magnesium sulfate in distilled water]. Each rat was fully perfused with saline and its total pituitary and adrenal glands (pooled left and right) were rapidly removed (less than 60 s).

### 4.4. Procedure for Protein and Enzymatic Assays

Tissue samples were homogenized in an hypoosmolar medium (10 mmol/L HCl-Tris buffer, pH 7.4) and ultracentrifuged at 100,000× *g* for 30 min at 4 °C. To obtain the particulate fraction, the pellets were re-homogenized in a HCl-Tris buffer (pH 7.4) and 1% Triton X-100 to solubilize membrane proteins. After centrifugation (100,000× *g*, 30 min, 4 °C), the protein level and activity of membrane-bound enzymes were measured in triplicate in the supernatants. To ensure complete recovery of activity, the detergent was removed from the medium by adding adsorbent polymeric Bio-Beads SM-2 (Sigma-Aldrich, St Louis, MO, USA)) (100 mg/mL) and shaking the samples for 2 h at 4 °C. The activity of membrane-bound aminopeptidases, measured as glutamyl- (GluAP), alanyl- (AlaAP) and cystinyl-aminopeptidase (CysAP), was determined fluorometrically using the arylamide derivatives, glutamyl-, alanyl- and cystinyl-β-naphthylamide, as substrates as previously described [23]. Briefly, GluAP was determined using Glu-β-naphthylamide as a substrate: 10 mL of each supernatant was incubated for 120 min at 37 °C with 1 mL of the substrate solution (2.72 mg/100 mL Glu-β-naphthylamide, 10 mg/ 100 mL bovine serum albumin (BSA), 10 mg/100 mL dithiothreitol (DTT) and 0.555 g/100 mL CaCl_2_ in 50 mmol/L HCl-Tris, pH 7.4). AlaAP and CysAP were measured using Ala- or Cys-β-naphthylamide as substrates, such that 10 mL of each supernatant and plasma were incubated for 30 min at 25 °C with 1 mL of the substrate solution, i.e., 2.14 mg/100 mL of Ala-β-naphthylamide or 5.53 mg/100 mL of Cys-β-naphthylamide, 10 mg/100 mL BSA and 10 mg/100 mL DTT in 50 mmol/L of phosphate buffer (pH 7.4 for AlaAP) and 50 mmol/L HCl-Tris buffer (pH 6 for CysAP). The reactions were terminated by addition of 1 mL of 0.1 mol/L of acetate buffer, pH 4.2. The amount of β naphthylamine released as a result of the enzymatic activity was measured fluorometrically at a 412 nm emission wavelength with an excitation wavelength of 345 nm. Proteins were quantified in triplicate using the method of Bradford [24] with BSA as a standard. Specific membrane-bound aminopeptidase activities were expressed as a nmol of the corresponding substrate hydrolyzed per minute per milligram of protein. Fluorogenic assays were linear with respect to time of hydrolysis and protein content.

### 4.5. Statistical Analysis

To analyze differences between the groups we used a two-way analysis of variance. *P* values below 0.05 were considered significant. To examine intra-gland and inter-gland correlations between aminopeptidase activities in each group of WKY or SHR studied, Pearson’s coefficient of correlation was computed. Computations were performed using SPSS 13.0 (Chicago, IL, USA) and STATA 9.0 (STATA Corp, College Station, TX, USA). *P* values below 0.05 were considered significant.

## 5. Conclusions

The mechanisms involved in the development of hypertension and the strategies to counteract and reverse it are being widely analyzed. How the various metabolic conditions derived from antihypertensive drug treatments are affecting the treatment efficacy are still far from being fully elucidated. The present results reflect additional neuroendocrine differences between normo- and hypertensive baseline states as well as additional functional consequences of the pharmacological treatments on the pituitary-adrenal axis.

## Figures and Tables

**Table 1:** Positive or negative significant correlations between the aminopeptidase activities analyzed in the pituitary and adrenal glands of each group of treatments in WKY and SHR.

WKY			SHR		
Correlation	r	*p*	Correlation	r	*p*
Control					
PT AlaAP vs.PT CysAP	+0.879	0.0007	*PT CysAP* vs. *AD GluAP*	−*0.751*	*0.03*
			AD AlaAP vs. AD CysAP	+0.885	0.003
Captopril					
PT AlaAP vs. PT CysAP	+0.744	0.01	PT CysAP vs. AD AlaAP	+0.817	0.01
*AD CysAP* vs. *AD GluAP*	−*0.726*	*0.01*	PT CysAP vs. AD CysAP	+0.812	0.01
			PT CysAP vs. AD GluAP	+0.737	0.03
			AD AlaAP vs. AD CysAP	+0.949	0.0003
			AD AlaAP vs. AD GluAP	+0.830	0.01
			AD CysAP vs. AD GluAP	+0.742	0.03
Propranolol					
PT AlaAP vs. PT CysAP	+0.631	0.03	PT AlaAP vs. PT CysAP	+0.785	0.02
PT AlaAP vs. PT GluAP	+0.768	0.005	AD AlaAP vs. AD CysAP	+0.847	0.007
PT AlaAP vs. AD CysAP	+0.624	0.03	AD CysAP vs. AD GluAP	+0.712	0.04
AD AlaAP vs. AD CysAP	+0.665	0.02			
AD AlaAP vs. AD GluAP	+0.876	0.0004			
AD CysAP vs. AD GluAP	+0.689	0.01			
L-NAME					
PT AlaAP vs. PT CysAP	+0.721	0.02	AD AlaAP vs. AD CysAP	+0.909	0.001
PT AlaAP vs. PT GluAP	+0.834	0.005			
*PT CysAP* vs. *AD GluAP*	−*0.731*	*0.02*			

## Data Availability

The data presented in this study are available on request from the corresponding author.

## Abbreviations

AD	adrenals
ADH	antidiuretic hormone
AlaA	alanine aminopeptidase
Ang	angiotensin
AT_4_	AT_4_ receptor
CAP	captopril
CysAP	cystine aminopeptidase
CT	control
GluAP	glutamate aminopeptidase
IRAP	insulin-regulated aminopeptidase
LN	L-NAME
PRO	propanolol
PT	pituitary
RAS	Renin-angiotensin system
SHR	spontaneously hypertensive rats
WKY	Wistar–Kyoto

## References

[1] Ramírez-Sánchez M., Prieto I., Wangensteen R., Banegas I., Segarra A.B., Villarejo A.B., Vives F., Cobo J., de Gasparo M. (2013). The renin-angiotensin system: New insight into old therapies. Curr. Med. Chem..

[2] de Gasparo M., Speth R.C., Baltatu O.C., Vanderheyden P. (2013). Brain RAS: Hypertension and beyond. Int. J. Hypertens..

[3] Arendse L.B., Danser A.H.J., Poglitsch M., Touyz R.M., Burnett J.C., Llorens-Cortes C., Ehlers M.R., Sturrock E.D. (2019). Novel therapeutic approaches targeting the renin-angiotensin system and associated peptides in hypertension and heart failure. Pharmacol. Rev..

[4] Llorens-Cortes C., Touyz R.M. (2020). Evolution of a new class of antihypertensive drugs: Targeting the brain renin-angiotensin system. Hypertension.

[5] Ramírez M., Prieto I., Alba F., Vives F., Banegas I., de Gasparo M. (2008). Role of central and peripheral aminopeptidase activities in the control of blood pressure: A working hypothesis. Heart Fail. Rev..

[6] Albiston A.L., McDowall S.G., Matsacos D., Sim P., Clune E., Mustafa T., Lee J., Mendelsohn F.A., Simpson R.J., Connolly L.M. (2001). Evidence that the angiotensin IV (AT(4)) receptor is the enzyme insulin-regulated aminopeptidase. J. Biol. Chem..

[7] Prieto I., Villarejo A.B., Segarra A.B., Wangensteen R., Banegas I., de Gasparo M., Vanderheyden P., Zorad S., Vives F., Ramírez-Sánchez M. (2015). Tissue distribution of CysAP activity and its relationship to blood pressure and water balance. Life Sci..

[8] Stragier B., De Bundel D., Sarre S., Smolders I., Vauquelin G., Dupont A., Michotte Y., Vanderheyden P. (2008). Involvement of insulin-regulated aminopeptidase in the effects of the renin-angiotensin fragment angiotensin IV: A review. Heart Fail. Rev..

[9] Marc Y., Llorens-Cortes C. (2011). The role of the brain renin-angiotensin system in hypertension: Implications for new treatment. Prog. Neurobiol..

[10] Zhang L., Edwards D.G., Berecek K.H. (1996). Effects of early captopril treatment and its removal on plasma angiotensin converting enzyme (ACE) activity and arginine vasopressin in hypertensive rats (SHR) and normotensive rats (WKY). Clin. Exp. Hypertens..

[11] Prieto I., Segarra A.B., de Gasparo M., Martínez-Cañamero M., Ramírez-Sánchez M. (2018). Divergent profile between hypothalamic and plasmatic aminopeptidase activities in WKY and SHR. Influence of beta-adrenergic blockade. Life Sci..

[12] Prieto I., Segarra A.B., Villarejo A.B., de Gasparo M., Martínez-Cañamero M.M., Ramírez-Sánchez M. (2019). Neuropeptidase activity in the frontal cortex of Wistar-Kyoto and spontaneously hypertensive rats treated with vasoactive drugs: A bilateral study. J. Hypertens..

[13] Segarra A.B., Prieto-Gomez I., Banegas I., Martínez-Cañamero M., Luna J.D., de Gasparo M., Ramírez-Sánchez M. (2019). Functional and neurometabolic asymmetry in SHR and WKY rats following vasoactive treatments. Sci. Rep..

[14] Prieto I., Villarejo A.B., Segarra A.B., Banegas I., Wangensteen R., Martinez-Cañamero M., de Gasparo M., Vives F., Ramírez-Sánchez M. (2014). Brain, heart and kidney correlate for the control of blood pressure and water balance: Role of angiotensinases. Neuroendocrinology.

[15] Segarra A.B., Prieto I., Banegas I., Martínez-Cañamero M., de Gasparo M., Ramírez-Sánchez M. (2021). Blood pressure correlates asymmetrically with neuropeptidase activities of the left and right frontal cortices. Symmetry.

[16] Clough D.P., Hatton R., Keddie J.R., Collis M.G. (1982). Hypotensive action of captopril in spontaneously hypertensive and normotensive rats. Interference with neurogenic vasoconstriction. Hypertension.

[17] Bhagat B.D. (1979). Mechanism of the antihypertensive effect of propranolol. Gen. Pharmacol..

[18] Pechanova O., Vrankova S., Cebova M. (2020). Chronic L-Name-treatment produces hypertension by different mechanisms in peripheral tissues and brain: Role of central eNOS. Pathophysiology.

[19] Eshima K., Hirooka Y., Shigematsu H., Matsuo I., Koike G., Sakai K., Takeshita A. (2000). Angiotensin in the nucleus tractus solitarii contributes to neurogenic hypertension caused by chronic nitric oxide synthase inhibition. Hypertension.

[20] Priviero F.B., Teixeira C.E., Claudino M.A., De Nucci G., Zanesco A., Antunes E. (2007). Vascular effects of long-term propranolol administration after chronic nitric oxide blockade. Eur. J. Pharmacol..

[21] Domínguez-Vías G., Aretxaga-Maza G., Prieto I., Luna J.D., de Gasparo M., Ramírez-Sánchez M. (2017). Diurnal opposite variation between angiotensinase activities in photo-neuro-endocrine tissues of rats. Chronobiol. Int..

[22] Slaiby J.M., Ricci M.A., Gadowski G.R., Hendley E.D., Pilcher D.B. (1994). Expansion of aortic aneurysms is reduced by propranolol in a hypertensive rat model. J. Vasc. Surg..

[23] Ramírez M., Prieto I., Banegas I., Segarra A.B., Alba F. (2011). Neuropeptidases. Methods Mol. Biol..

[24] Bradford M.M. (1976). A rapid and sensitive method for the quantitation of microgram quantities of protein utilizing the principle of protein-dye binding. Anal. Biochem..

